# Nursing care factors influencing patients' outcomes in the intensive care unit: Findings from a rapid review

**DOI:** 10.1111/ijn.12962

**Published:** 2021-05-17

**Authors:** Matteo Danielis, Anne Lucia Leona Destrebecq, Stefano Terzoni, Alvisa Palese

**Affiliations:** ^1^ Department of Clinical Sciences and Community Health University of Milan Milan Italy; ^2^ School of Nursing, Department of Biomedical Sciences for Health University of Milan Milan Italy; ^3^ School of Nursing San Paolo Hospital Milan Italy; ^4^ School of Nursing, Department of Medical and Biological Sciences Udine University Udine Italy

**Keywords:** adult patient, intensive care unit, nursing‐sensitive outcomes, nursing interventions

## Abstract

**Aims:**

To examine the nursing care factors investigated regarding their influence on outcomes of critically ill patients.

**Background:**

A large number of studies have considered patients' outcomes as sensitive to nursing practice in intensive care unit environments. However, no summary of nursing factors influencing these outcomes has been provided.

**Design:**

Rapid review, following the seven‐stage process outlined by Tricco and colleagues.

**Data Sources:**

Articles published up to March 2020 were identified in MEDLINE (via PubMed), Cumulative Index to Nursing and Allied Health Literature (CINAHL) and Scopus databases.

**Review Methods:**

Eligibility of studies was first assessed at the title and abstracts level. Study inclusion was then established by two researchers by analysing the full texts.

**Results:**

A total of 93 studies were included, with a total of 21 nursing care factors documented. At the *structural* level, nursing factors have been investigated at the organizational and at the personnel level. At the *process* level, nurse‐led programmes, independent nursing interventions and nurse behaviours have been investigated to date.

**Conclusion:**

The set of nursing factors that emerged can be used in future research to improve poorly developed areas and to accumulate further evidence through additional studies, both at managerial and practice levels.

## Summary statement

What is already known about this topic?

- Thirty‐five nursing‐sensitive outcomes capable of being used to measure the quality of care in the intensive care unit have been identified to date.
- Several nursing care factors in structural and process dimensions have been documented as having an influence on the outcomes of critically ill patients.

What this paper adds:

- Many interventions have been assessed in relation to nursing outcomes, more often at the process than at structural levels.
- Specifically, a total of 21 nursing factors have been studied to date in the context of intensive care units, divided into structure (organizational and personnel) and process (nurse‐led programmes, independent nursing interventions and behaviours) dimensions.

The implications of this paper

- The set of nursing factors that emerged can be considered as a basis for further research, especially regarding poorly developed areas.
- Emergent nursing care factors can be used as a blueprint to design and develop educational programmes both at under‐ and postgraduate levels.
- At the managerial levels, both structure and process dimensions of nursing care are capable of affecting outcomes and could be used to inform decision‐making.

## 1 INTRODUCTION

The literature regarding the relationship between nursing care and patient outcomes has increased in recent years (Myers et al., 2018) with the intent to promote high quality of care and to demonstrate the value of nursing care (Di Giulio et al., 2019; Salmond & Echevarria, 2017). Theoretically, the quality of care has been conceptualized by Donabedian (1988) as composed of three distinct dimensions or levels, namely, (1) *structure*, consisting of the setting where the care is provided and covering organizational variables; (2) *process*, including all interventions as performed by healthcare professionals; and (3) *outcome*(s), as the change(s) measured in the patients' health status.

In the specific field of nursing care, ‘structure’ has been reported as including the nurse‐to‐patient ratio (Blot et al., 2011), whereas ‘process’ has been described as including interventions performed independently or under physicians' prescription (e.g. weaning from mechanical ventilation) (Al Ashry et al., 2016). With regard to outcomes, in line with Doran's (2011) definition, a nursing‐sensitive outcome (NSO) has been defined as any change in a patient's health state, behaviour or perception, associated with or sensitive to the nursing care received (e.g. reduced pressure injury with preventive nursing care) (Karadag et al., 2017). Recently, a total of 35 NSOs have been identified as appropriate in intensive care unit (ICU) contexts (Danielis et al., 2019). According to their features, these outcomes have been categorized as (1) clinical (general health and goal assessment and monitoring), (2) functional (psychosocial and physical dimensions), (3) safety (critical incidents and healthcare‐associated infections) and (4) perceptive (experience of being in ICU). However, NSOs have been documented to vary across patients and settings (Danielis et al., 2019; Myers et al., 2018) and also according to nursing care factors.

Several nursing care factors expressing the structural and the process dimensions have been documented as having an influence on NSOs (Myers et al., 2018). Among the first, higher patient mortality, infections, post‐operative complications and missed nursing activities have been associated with lower levels of nurse staffing (Cho et al., 2019; Lee et al., 2017; Penoyer, 2010). Several studies have also documented the role of the work environments and that of staff workloads in affecting patients' safety (Kelly et al., 2013; Ulrich et al., 2019). Additional research has shown that some structural dimensions of nursing care are associated with outcomes among critically ill patients in open or closed ICUs and the availability of clinical nurse specialists (Checkley et al., 2014). Nurses with advanced competence in ICU have been documented to enhance patient satisfaction and to decrease mortality rates and the length of stay (LOS), thus saving on the costs associated with care (Woo et al., 2017).

With regard to the process dimensions, available studies have documented that rounding practices (e.g. daily meetings between physician and charge nurse) and the use of protocols (e.g. regarding mobility and delirium management) have been associated with lower ICU mortality (Checkley et al., 2014). Moreover, an early and timely start of enteral feeding (Orinovsky & Raizman, 2018) and intensive glycaemic control (Khalaila et al., 2011), both considered as nursing care interventions, have also been documented to improve NSOs.

Despite the rich debate, no reviews mapping those nursing care factors investigated for their influence on NSOs have been published in the ICU field. Therefore, no summaries about the state of the science in intervention studies have been produced, nor are there any critical reflections on the most studied or understudied nursing care factors. Among the various reasons for this lack of evidence, one may be due to the predominance of multidisciplinary processes in the ICU settings that lead to diverse implications of professional and research approaches. Given that multidisciplinary interventions prevail, firstly, nursing care is sometimes overshadowed in its capacity to affect NSOs (Needleman, 2017). Secondly, intervention studies in the field have a high degree of complexity, given that the contribution of nursing care is not easily discernible from other factors affecting patient outcomes (Myers et al., 2018). The main intent of this review is to overcome these challenges by providing a summary of the state of the art in the field of the nursing care factors affecting NSOs in ICU.

### 1.1 Aim

The main aim of the study was to summarize the structural and process nursing care factors that have been studied to date and their influence on the outcomes of critically ill patients.

## 2 REVIEW METHODS

### 2.1 Study design

A rapid review, as a form of knowledge synthesis, is capable of providing timely information (O'Leary et al., 2017) and was performed in March 2020. As this approach uses a streamlined systematic review methodology, the present study design was conducted as a pragmatic approach to provide information to ICU clinical nurses, managers and decision‐makers (O'Leary et al., 2017). According to the methodological process inspired by Tricco et al. (2017) and then further developed by Langlois et al. (2019), the following seven‐stage process was performed: (1) needs assessment and topic selection, (2) study development, (3) literature search, (4) screening and study selection, (5) data extraction, (6) risk‐of‐bias assessment and (7) knowledge synthesis. In line with the study design and aims, no quality appraisal of the studies was performed, and a selective process of data extraction was applied.

### 2.2 Needs assessment and topic selection

The primary need was to map the nursing care factors associated with NSOs, with the intent of summarizing those factors investigated to date and highlighting those in need of further research investments. Thus, the review question was: What nursing care factors at the structure and the process levels, capable of influencing outcomes of critically ill adult patients cared for in ICU, have been studied to date?

### 2.3 Study development

Following the Preferred Reporting Items for Systematic Reviews and Meta‐Analysis (PRISMA) guidelines (Moher et al., 2009), the search was performed according to population, intervention and outcome (PIO) statements (Eriksen & Frandsen, 2018) as follows: (P) population, critically ill adult patients admitted and cared for in ICU; (I) intervention(s), any nursing care factors at the structure and process levels of care delivered to a patient (Donabedian, 1988); and (O) outcome(s), any outcome influenced by nursing care factors in ICU.

### 2.4 Literature search

The MEDLINE (via PubMed), the Cumulative Index to Nursing and Allied Health Literature (CINAHL), and the Scopus databases, as well as the grey literature, were searched up to March 2020. The authors set this time with the intention of including those nursing factors documented before the COVID‐19 pandemic caused by SARS‐CoV‐2 (Cucinotta & Vanelli, 2020). Consequently, the search strategy combined terms from three main themes: (1) ‘Intensive Care Units’[Mesh] OR ‘Critical Illness’[Mesh] OR ‘Critical Care’[Mesh] OR ‘Critical Care Nursing’[Mesh] OR ‘Critically ill patient’; (2) ‘Nursing’[Mesh] OR ‘Nursing Care’[Mesh] OR ‘Contribution of nursing care’ OR ‘Nursing interventions’; and (3) ‘Patient Outcome Assessment’[Mesh] OR ‘Outcome Assessment (Health Care)’[Mesh] OR ‘Treatment Outcome’[Mesh] OR ‘Critical Care Outcomes’[Mesh] OR ‘Outcome Measures’. All these terms and free‐text words were combined into search strings with the Boolean operator ‘AND’.

### 2.5 Screening and study selection

Studies were included when they (1) assessed NSO(s) as associated with nursing factors at the structural and process levels, (2) were performed in adult (≥18 years old) ICU patients, (3) as primary (e.g. randomized control trials) and secondary study designs (e.g. systematic reviews) and (4) published in English. Therefore, those studies concerning (1) the paediatric population (<18 years), (2) terminally ill patients, (3) settings other than ICU (e.g. recovery rooms), (4) not focused on the specific contribution of nursing care and (5) published in languages other than English were all excluded.

**FIGURE 1:**
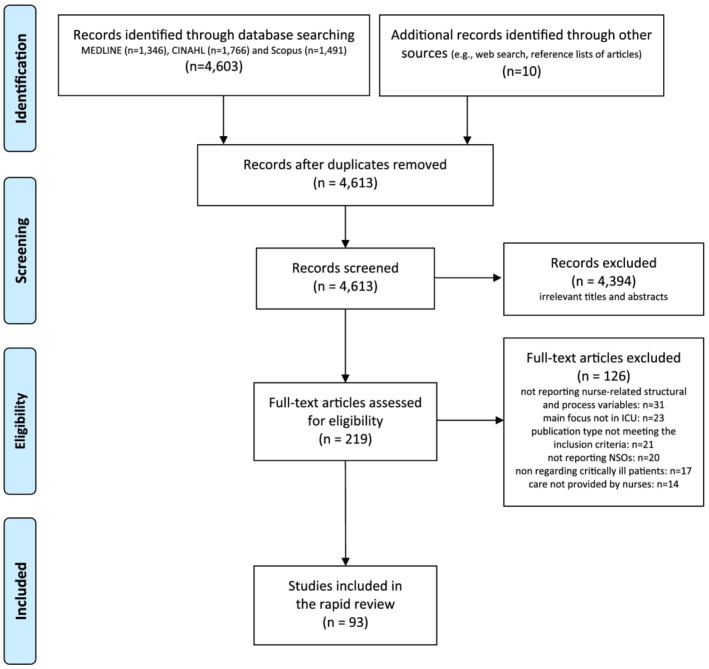
Review flow diagram (Moher et al., 2009)

In the first level of screening, the titles and abstracts of retrieved studies were evaluated for their eligibility against the inclusion criteria by two researchers (MD, AP) independently. A third reviewer was included to resolve disagreements, if any (AD). Then, an independent full‐text review was performed to determine if the studies meet the inclusion criteria. Also in this step, in case of doubt, a third researcher was involved. At the end of the process, 93 studies were retrieved, as reported in the PRISMA flow diagram (Moher et al., 2009) (Figure 1).

### 2.6 Data extraction

The following data were extracted from each included study and reported in a Microsoft Excel spreadsheet: (1) author(s), journal, publication year and country; (2) study design, type of ICU (e.g. general or specialized), the study aims and participant profiles; (3) nursing care factors(s) as evaluated in their association with NSOs—each nursing care factor was briefly described together with the outcomes linked to it; and (4) key findings. The full grid is available as Table S1 on the website. Secondary research designs (e.g. systematic reviews) were also included and discussed as a single study. This process was completed by the first author with the supervision of the last author.

According to the nature of the rapid review, selected studies were considered to scope the available literature rather than to evaluate the effects of specific nursing care factors. No quality appraisal of the studies was performed.

### 2.7 Risk of bias assessment

The following strategies were applied to prevent bias: (1) The review team shared each step of the study inclusion and exclusion process; (2) MEDLINE (via PubMed), CINAHL and Scopus were accessed as major scientific databases; (3) the data extraction was performed at by at least two reviewers; and (4) the summary table, as well as the narrative synthesis, were both reviewed by a third independent researcher.

### 2.8 Knowledge synthesis

With regard to the rapid review question, a narrative synthesis of the available studies was performed. Nursing care factors were first categorized according to their common organizational or clinical significance (e.g. hours of nursing care per day, music therapy and care bundle compliance) by two researchers (MD, AP), one an expert in ICU care (MD). Then, these factors were classified according to the Donabedian model dimensions (Donabedian, 1988), namely, structure and process. Therefore, by combining the first and the second categorization, the following classifications emerged:

- Structure dimension: factors at (1) organizational level, which included organizational and workplace culture, and (2) nursing staff level, embracing all metrics for measuring nursing staff characteristics.
- Process dimension: factors of (3) nurse‐led programmes, understood as all interventions carried out by nurses, but shared and scheduled with a physician; (4) nurses' independent interventions, which refer to interventions planned and performed according to the nursing role; and (5) nurse behaviours, understood as the health‐promoting behaviours of nursing staff.

The categorization of the studies is reported in Table 1.

**TABLE 1:** Study categorization

Donabedian model component (Donabedian, 1988)	Categorization	Nursing care factor with its definition^a^	Study example
Structure	Organizational level	*Work environment*, as the organizational characteristics of the workplace that facilitate or confine nursing care practice	Patients in critical care units with better nurse work environments experienced 11% lower odds of 30‐day mortality than those in worse nurse work environments (Kelly et al., 2014)
		*Magnet hospital properties*, as nurse participation in hospital management, nursing foundations for quality of care, nurse manager abilities, staffing and resource adequacy, shared decision‐making between RN and MD	The associations found between hospital profitability and patient outcomes were mixed. In the CAUTI, VAP and decubiti models, there were significant positive relationships; hospitals with the lowest profit margin had less adverse outcomes than profitable ones (*P* < 0.05). The effects of magnet accreditation were not consistent (Stone et al., 2007)
		*Communication RN* vs. *MD*, as a mix of four core elements: openness, timeliness, accuracy and understanding	Timeliness of communication was inversely related to pressure ulcers (*r* = −0.38; *P* = 0.06), and workplace empowerment and scores on the Acute Physiology and Chronic Health Evaluation III were positive predictors of ventilator‐associated pneumonia (*R* ^2^ = 0.36; *P* = 0.005) (Manojlovich et al., 2009)
		*Material availability*, resources such as earplugs, eye masks and pressure redistributing mattresses	Seven studies assessed the effect of using the earplug or eye mask intervention on sleep in the ICU. The results for the intervention groups showed a beneficial impact (*P* < 0.05) for increased REM sleep and decreased REM latency in two of the six studies (Alway et al., 2013)
	Personnel level	*Nurse staffing*, as hours of nursing care per patient day, staff mix, skill mix and nurse‐to‐patient ratio	In this study that included 27 372 ICU patients discharged from 42 tertiary and 194 secondary hospitals, every additional patient per RN was associated with a 9% increase in the odds of dying (OR = 1.09, 95% CI = 1.04–1.14) (Cho et al., 2019)
		*Nurse specialist*, a nurse with a formal recognition of specialized knowledge, skills and clinical practice experience	The presence of a certified nurse specialist in critical care was associated with lower ICU mortality (OR = 0.52, 95% CI = 0.36–0.73, *P* < 0.001) and fewer patients receiving mechanical ventilation in the ICU (OR = 0.20, 95% CI = 0.15–0.26, *P* < 0.001) (Fukuda et al., 2020)
		*Nurse experience*, as working experience in years	Unplanned extubations occurred more frequently in the care of nurses with less experience, whereas experienced nurses (≥4 working years) encountered unplanned extubations less frequently (Yeh et al., 2004)
Process	Nurse‐led programmes	*Early mobility programmes*, as early mobility interventions from a dedicated mobility team	The 66 patients who received the mobility intervention had significantly fewer falls, ventilator‐associated events, pressure ulcers and CAUTIs than the 66 patients in the routine care group. The mobility group also reported lower hospital costs, fewer delirium days, lower sedation levels and improved functional independence (Fraser et al., 2015)
		*Checklists, algorithms and specific assessment tool*, as a predefined instrument to achieve standardization of processes	Following a feeding protocol, enteral nutrition started significantly earlier (28 ± 20 vs. 47 ± 34 h, *P* < 0.001), within 24 h in 64% vs. 25% (*P* < 0.0001); and for each of the first 5 days, the proportion of patients meeting their nutritional goal was significantly higher (Friesecke et al., 2014)
		*Family participation in patient care*, as promotion of family access to patient by effective planning of routine care	Patients receiving nurse‐facilitated family participation demonstrated better psychological recovery and wellbeing than the control group at 4, 8 and 12 weeks after admission to a critical care setting (Black et al., 2011)
		*Educational programmes*, as consistent approach to quality patient care, thereby resulting in less variability by sharing research‐based information	The intervention group who received the clinical nursing practice guideline had significantly shorter starting time of EN and a reduced duration of mechanical ventilator than those in the control group (*P* < 0.001) (Koontalay et al., 2020)
		*Reality orientation nursing programme*, as cognition‐orientated technique for patients with memory loss and spatial and temporal disorientation	Results from this quasi‐experimental study indicated that patients who received the reality orientation programme had a higher mean of the Glasgow Outcome Scale Extended than those receiving the usual care, despite that the groups differed significantly (*P* = 0.01) in post‐traumatic amnesia duration (Langhorn et al., 2015)
	Nurse's independent interventions	*Music therapy*, as complementary treatment in a therapeutic context	In the music group, there were statistically significant reductions (*P* = 0.001) in heart rate, respiratory rate and oxygen saturation than the control participants at 45 minutes (Chan et al., 2006)
		*Massage*, as non‐pharmacological hand activity with different techniques	In eight of 12 randomized control studies, there was a high level of evidence of favourable effects with respect to improvements in vital signs and a reduction in pain and anxiety (Jagan et al., 2019)
		*Relaxation and guided imagery*, as a cognitive‐behavioural technique in which the patient employs a mental process that uses images to alter a physical/emotional state	Among the 60 randomized critically ill adults in the sample, the intervention group experienced significant decreases in the incidence (*P* = 0.003) and ratings of pain (*P* < 0.001), systolic arterial pressure (*P* < 0.001), anxiety (*P* = 0.01) and improved quality of sleep (*P* = 0.02) (Papathanassoglou et al., 2018)
		*Body positioning*, as current positioning practices and degree of bed elevation	Intracranial pressure decreased with supine head of bed 45° (*P* < 0.01) and knee elevation, head of bed 30° and 45° (*P* < 0.05) and increased (*P* < 0.05) with right and left lateral head of bed 15°. Haemodynamic parameters were similar in the various positions (Ledwith et al., 2010)
		*Therapeutic touch*, as non‐pharmacological intervention indicates the use of hands on the patient's body	Repeated measures analysis of variance indicated no significant increases or decreases in any of the physiologic variables measured between pre‐, during and post‐time segments for therapeutic touch. The most frequently occurring words and phrases to describe the subjects' feelings were warmth, relaxation, tingling, calmness, sleepiness and the sensation of falling asleep (Cox & Hayes, 1999)
		*Aromatherapy*, as essential oils from plants administered via inhalation, massages and orally	Comparison of the Pittsburgh Sleep Quality Index and the Beck Anxiety Inventory scores at the patient's levels in the control and intervention groups before and after the intervention showed statistically significant differences in the change in favour of the intervention group (*P* < 0.05) (Karadag et al., 2017)
		*Cold application*, as ice therapy on an injured area	Patients in the cold group had significantly lower pain intensity than the placebo group. The application of cold prolonged the length of time until analgesics were needed after the chest tube removal (Demir et al., 2010)
	Nurse's behaviours	*Care bundle compliance*, as the multidisciplinary approach to patient care based upon a set of evidence‐based activities	Implementation of the bundle decreased average patient hospital length of stay by 1.8 days, reduced the length of mechanical ventilation by an average of 1 day and established a baseline delirium prevalence of 19% over a 3‐month time period (Kram et al., 2015)
		*Night‐time care routine interactions*, as the major environmental factor affecting the sleep of critically ill patients	Out of eight night‐time care routine interactions, only one (post‐operative exercises) was significantly associated to sleep variables (*r* > 0.40, *P* < 0.05) (Casida et al., 2018)

Abbreviations: CAUTI, catheter‐associated urinary tract infections; CI, confidence interval; EN, enteral nutrition; ICU, intensive care unit; MD, medical doctor; OR, odds ratio; REM, rapid eye movement; RN, registered nurse; VAP, ventilator‐associated pneumonia.

^a^ These definitions were developed by a combination of a priori knowledge of the research subject and a content analysis of the included studies.

## 3 RESULTS

### 3.1 Literature synthesis

As reported in Table 2, most studies (*n* = 74, 79.6%) were published in nursing journals. The earliest study was published in 1999, and more than two‐thirds of the articles (*n* = 72; 77.4%) were published after 2010. Studies were mainly authored in the United States and Canada (*n* = 36; 38.7%) and included mainly general (*n* = 68, 73.1%) ICU settings. With regard to study methods, 51 (54.8%) were observational in design, including cross‐sectional, case–control, prospective and retrospective cohort designs.

**TABLE 2:** Summary of study characteristics

Study characteristic	Number of studies (*n* = 93) *n* (%)
Journal source	
Nursing	74 (79.6)
Medical	19 (20.4)
Year of publication	
From 1999 to 2009	21 (22.6)
From 2010 to 2020	72 (77.4)
Continent	
US and Canada	36 (38.7)
Asia	19 (20.4)
Europe	17 (18.2)
Australia and New Zealand	10 (10.8)
Middle East	9 (9.7)
Central and South America	2 (2.2)
Setting (ICU type)	
General	68 (73.1)
Medical	8 (8.6)
Cardiovascular	7 (7.5)
Medical and surgical	6 (6.5)
Neurological	4 (4.3)
Study design	
Observational	51 (54.8)
Experimental and quasi‐experimental	30 (32.3)
Literature review	11 (11.8)
Mixed‐method	1 (1.1)

Abbreviations: ICU, intensive care unit; US, United States.

### 3.2 Nursing factors investigated to date

As reported in Table 3, a total of 21 nursing care factors measured against the 35 NSOs (Danielis et al., 2019) have been studied to date. Among them, early mobility programmes (*n* = 16, 45.7%) have been largely studied as being able to affect NSOs, followed by the use of algorithms, checklists and specific assessment tools (*n* = 14, 40.0%), nurse staffing (*n* = 12, 34.2%) and compliance with care bundles (*n* = 11, 31.4%). However, nurse orientation programmes and night‐time care routine interactions were studied only once, each regarding their influence on NSOs. According to Donabedian's (1988) model, the most frequently studied nursing care factors focus on the process dimension (*n* = 78, 65.0%), followed by the structure dimension (*n* = 42, 35.0%).

**TABLE 3:**
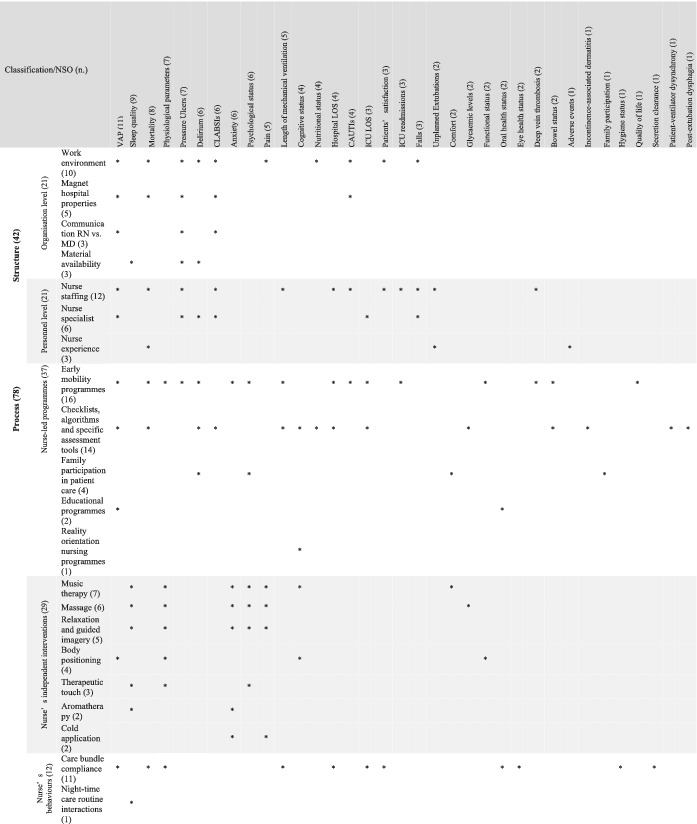
Structure and process nursing care factors studied to date regarding their influence on NSOs (*n* = 93 studies)

Abbreviations: CAUTIs, catheter‐associated urinary tract infections; CLABSIs, central line‐associated bloodstream infections; ICU, intensive care unit; LOS, length of stay; MD, medical doctor; NSOs, nursing‐sensitive outcomes; RN, registered nurse; VAP, ventilator‐associated pneumonia. [Correction added on 15 June 2021, after first online publication: table 3 reformatted as an image for clarity and legibility.]

### 3.3 Structure dimension

As reported in Table 3, in terms of the structure dimension, nursing factors influencing NSOs have been investigated both at an organizational level and at the personnel level.

Factors at the organizational level were widely studied with regard to their influence on 21 NSOs. Specifically, the work environment has been documented in terms of its influence on 10 NSOs (e.g. healthcare‐associated infections and falls); magnet hospital properties have been studied against five outcomes (e.g. catheter‐associated urinary tract infections and mortality), followed by nurse–physician communication measured regarding three NSOs (e.g. pressure ulcers and central line‐associated bloodstream infections), whereas material availability has been studied for its ability to influence the occurrence of pressure ulcers, delirium and the sleep quality.

Factors at the personnel level have been investigated with regard to 21 NSOs; specifically, nurse staffing has been studied regarding 12 (e.g. the length of mechanical ventilation and ICU readmissions), followed by the nurse specialist role explored in terms of its effect regarding six (e.g. delirium and falls occurrences) and nurse experience regarding three NSOs (e.g. unplanned extubations and adverse events).

### 3.4 Process dimension

As reported in Table 3, studies that evaluated the nursing care factors at the process dimension included nurse‐led programmes, nurses' independent interventions and nurse behaviours.

Nurse‐led programmes were described regarding their effect on 37 NSOs. These included, particularly, early mobility programmes, studied in relation to 16 NSOs (e.g. hospital LOS and patient–ventilator dyssynchrony), followed by checklists, algorithms and specific assessment tools in relation to 14 NSOs (e.g. nutritional and bowel status). Then, in order of frequency, programmes facilitating family participation in patient care were studied for their influence on four NSOs (e.g. comfort), educational programmes for two NSOs (ventilator‐associated pneumonia and oral health status) and a reality orientation programme for cognitive status.

Independent nursing interventions have been investigated in relation to 29 NSOs. The most reported factor was music therapy, influencing seven NSOs (e.g. comfort and pain), followed by massage interventions focused on six NSOs (e.g. sleep quality and physiological parameters), relaxation and guided imagery for five NSOs (e.g. anxiety and pain), body positioning in relation to four NSOs (e.g. ventilator‐associated pneumonia and physiological parameters), therapeutic touch for three NSOs (e.g. psychological status), aromatherapy and cold application for two outcomes (e.g. sleep quality and pain, respectively).

Lastly, nurse behaviours have been studied in relation to 12 NSOs. The most reported factor was care bundle compliance, which has been measured regarding its influence on 11 NSOs (e.g. patient satisfaction and secretion clearance), followed by night‐time care routine interactions for their capacity to affect sleep quality.

## 4 DISCUSSION

A total of 93 studies emerged, mainly in nursing journals in a span of 20 years, with on average just over four articles per year. Most articles were published in the last 9 years, suggesting that intervention studies are attracting increased interest in the last years. In this regard, no comparison can be performed with other fields of nursing care because, to the best of our knowledge, no similar rapid review has been performed to date. Twenty‐one nursing care factors have been investigated to date, and according to the categorization applied (Donabedian, 1988), most were at the process level and fewer at the structural level.

### 4.1 Structure dimension

At the structure level, the nursing work environment and staffing characteristics were studied mainly in relation to similar outcomes such as the occurrence of ventilator‐associated pneumonia, pressure ulcers, central line‐associated bloodstream infections and mortality. Communication between nurses and physicians has been examined in relation to ventilator‐associated pneumonia, pressure ulcers and central line‐associated bloodstream infections. These factors have been largely studied also in other contexts as mediating NSOs, thus not directly influencing outcomes, although they have been documented as improving job satisfaction (Ulrich et al., 2019) and enabling nurses to improve their performance, the quality of their clinical surveillance and their compliance with aseptic techniques (Stone et al., 2007).

Within the structure dimension, regarding personnel‐level categorization, three factors have been investigated in relation to different NSOs. The numbers of nursing staff have been reported as hours of nursing care per patient day (Heslop & Lu, 2014), as staff skill mix (Sales et al., 2011) and as the nurse‐to‐patient ratio (Yeh et al., 2004). Nurse staffing has been considered regarding its influence on outcomes such as length of mechanical ventilation, hospital LOS, the occurrence of catheter‐associated urinary tract infections and others. Less emphasis has been devoted to date to the nurse specialist roles and experience. A recent meta‐analysis that included 35 studies published between 2006 and 2017 involving 175 755 patients showed that a higher nurse staffing level decreased the risk of in‐hospital mortality by 14% in specialist ICUs (Driscoll et al., 2018), suggesting the availability of a strong body of knowledge. In contrast, the types of specialty certifications, different degrees of advanced nursing practice, job descriptions and nurses' work experience of ICU seem to require further research with the intent of clarifying their contributions to patient outcomes and to inform policy development concerning the requirements in terms of the professional profile of ICU nurses.

### 4.2 Process dimension

In the process dimension (Donabedian, 1988), three different factors emerged. The most investigated were programmes led by nurses (e.g. educational programmes), followed by independent nursing interventions (e.g. music therapy) and those regarding nurse behaviours (e.g. care bundle compliance). Considering the programmes led by nurses, most studies reported the implementation of early mobility programmes and the use of checklists, algorithms and specific assessment tools to improve outcomes such as ventilator‐associated pneumonia and mortality rates. Nowadays, accumulating evidence is suggesting that nurse‐led approaches are more suitable, effective and cost‐saving for disease management (Klein et al., 2018; Orinovsky & Raizman, 2018). This is the case where, for example, nurse‐led weaning programmes led to a reduction in the length of mechanical ventilation (Kram et al., 2015). With regard to independent nursing interventions, the use of music therapy has been largely documented (Aktas & Karabulut, 2016), followed by massage, relaxation and guided imagery, body positioning, therapeutic touch, aromatherapy and cold application. Accordingly, these interventions were assessed in relation to different NSOs; however, the number of studies available was limited, possibly due to the prevailing multidisciplinary nature of the work care processes in the ICU (Marshall et al., 2017). Nurse behaviours, such as care bundle compliance, have been studied in relation to various outcomes including the incidence of ventilator‐associated pneumonia, preventing infections associated with care practices and patient mortality.

On the other hand, night‐time care routine interactions have been less investigated. This lack of studies can be interpreted along two lines of reasoning: (1) Measuring behaviours in clinical practice is challenging, as it requires a long engagement in the research process (Lambert & Housden, 2017), and (2) measuring the outcomes of violations in care delivery when strong recommendations are available is less important than understanding why and in which organizational condition nurses fail to maintain their compliance to bundles of care or good practice. In other words, although it is important to continue to study the relevance of this factor in relation to its implications for NSOs, the focus should be on the variables underlying poor compliance or on those that are able to maximize the quality of care delivered.

### 4.3 Implications of the findings

A total of 21 nursing factors have been assessed in relation to 35 ICU NSOs to date. Firstly, the set of nursing factors that emerged could be considered as the basis for further research, especially regarding those poorly investigated. Periodically assessing what interventions in relation to which outcomes have been studied could direct future research to fill in gaps as well as to explore similar interventions and outcomes, thus accumulating further evidence. Secondly, at the clinical level, having a map of the interventions assessed, as well as which outcomes were studied, could stimulate nurses' participation in scientific development of nursing knowledge, for instance, of interventions performed on a daily basis and not to date considered by researchers (Smith et al., 2016). Thirdly, the set of nursing care factors can be used as a *blueprint* to design and develop educational programmes both at the undergraduate and postgraduate levels. Moreover, as both structure and processes dimensions of nursing care are capable of affecting NSOs, this information might be useful to inform managerial decision‐making.

### 4.4 Strengths and limitations

This rapid review contributes new knowledge in summarizing the state of the science on nursing care factors influencing patient outcomes in ICU. An established rapid review method (Tricco et al., 2017) was performed; however, although its findings can inform researchers and clinicians on emergent issues (Langlois et al., 2019), these should be considered within the limitations of this review design, which does not specifically assess the study characteristics, specific ICU settings, patients or the effectiveness of the interventions. In accordance with O'Leary et al. (2017), the methodological quality of the studies included was not assessed. Secondly, although a systematic approach was followed according to the study design, some selection and information bias may have occurred, and some studies missed. Lastly, some reviews have been included with the intent of covering a broad spectrum of literature and intercepting all nursing care factors investigated to date. Some reviews might have analysed primary studies already included in this rapid review, thus introducing a potential bias regarding the duplicates.

## 5 CONCLUSION

This rapid review highlighted that, to date, a broad set of interventions has been assessed against the NSOs, with a greater number at the process levels and fewer at the structural dimension levels. Findings suggest that researchers are attracted mainly to modifiable variables with the intent to establish effective nursing care processes; however, structural variables are also capable of influencing patient outcomes. Overall, the set of nursing factors that emerged can be used as a map for researchers, educators, managers and clinicians in their various roles. Future studies should try to combine factors at the structural and process levels in their capacity to influence NSOs, given that, according to the findings, they have been investigated separately.

According to the findings, mobility programmes, the use of algorithms, checklists and specific assessment tools, appropriate nurse staffing and compliance with care bundles have been largely studied as they are able to affect the NSOs of critically ill patients. However, interventional studies aimed at evaluating the effectiveness of specific nursing care factors (e.g. body positioning, family involvement and educational programmes) are needed; similarly, nurse specialist roles and nurse experience in ICU should be fully documented with details on their capacity to affect patients' outcomes. Moreover, periodically repeating an assessment of the nursing care factors investigated in relation to NSOs might support the analysis of emerging trends.

## CONFLICT OF INTEREST

No conflict of interest has been declared by the authors.

## AUTHORSHIP STATEMENT

All authors had substantial contributions to (1) design of literature search (MD, AD and AP), (2) drafting the article (MD, AD and AP) or revising it critically for important intellectual content (MD, ST and AP) and (3) final approval of the version to be published (MD, AD, ST and AP).

## Supporting information


**Table S1** Data extraction process
