# Supplementary material for: Nursing care factors influencing patients' outcomes in the intensive care unit: Findings from a rapid review
Source: Int J Nurs Pract. 2021 May 17;28(2):e12962. doi: 10.1111/ijn.12962 (PMC9286446; doi:10.1111/ijn.12962)
Supplement: Supplementary file 1 — Table S1 Data extraction process [file IJN-28-0-s001.docx]

| **Supplementary Table 1** Data extraction process | | | | | | | |
| --- | --- | --- | --- | --- | --- | --- | --- |
| *First author, year* | *Country* | *Design* | *Setting* | *Aim* | *Sample inclusion criteria* | *Participant: profile* | *Nursing care intervention(s) as independent variable(s)* |
| Aktas, 2016 | Turkey | Randomised controlled trial | Cardiovascular ICU | To examine the effect of music therapy on pain, sedation and physiologic parameters during endotracheal suctioning of mechanically ventilated patients | Patients who were scheduled for a cardiothoracic surgery that met these inclusion criteria: ≥18 years old, intubated and needing endotracheal suctioning and in the level of wakefulness 2 or 3 according to Ramsay Sedation Scale | 66 patients  (33 experimental and 33 control) | Music therapy |
| Al Ashry, 2014 | Nebraska, US | Observational (before/after) | General ICU | To assess whether nursing compliance with filling an 18-item ICU checklist is associated with improvement in clinical outcomes in mechanically and nonmechanically ventilated ICU patients | A consecutive series of patients admitted to the ICU between December 2012, and June 2013 were included in this study | 349 ICU patients | Nurse-led ICU checklist |
| Almerud, 2003 | Sweden | Mixed method | General ICU | To ascertain whether music therapy had a measurable relaxing effect on patients who were temporarily on a respirator in an intensive care unit (ICU) and after completion of respirator treatment, investigate those patients’ experiences of the music therapy | Adults, intensive care patients who were temporarily in need of mechanical ventilation and whose condition was physically stable was included | 20 patients, ten patients were included in each group | Music therapy |
| Alm-Kruse, 2008 | Norway | Observational (before/after) | General ICU | To evaluate the existing degree of glycaemic control, and to implement SGC safely in our ICU through a nurse-led implementation of an algorithm for intensive insulin-therapy | All patients admitted during the 32 months of the study were enrolled | 494 patients of whom 210 had a length of stay in excess of 72 hours | Nurse-led algorithm |
| Alway, 2013 | Oregon, US | Literature review | General ICU | Review question: what effect do the interventions of earplugs and eye masks have on sleep and delirium outcomes in critically ill adults? | Key words included earplugs, eye masks, sleep quality, sleep deprivation, sensory overload, and delirium. The search was limited to original research studies | 7 studies retrieved | Earplugs and eye mask |
| Amaravadi, 2000 | Maryland, US | Cohort study | General ICU | To determine if having a night-time nurse-to-patient ratio of one nurse caring for one or two patients versus one nurse caring for three or more patients in the ICU is associated with clinical and economic outcomes | All adult patients discharged from Maryland hospitals from 1994 to 1998 with a primary procedure code for oesophageal resection were included | A total of 366 adult patient underwent oesophageal resection | Nurse-to-patient ratio |
| Andrews, 2014 | Virginia, US | Observational (before/after) | General ICU | To evaluate the implementation and effects of the Confusion Assessment Method for the Intensive Care Unit as a bedside assessment for delirium | Charts of all patients 18 to 89 years old admitted to the general ICU during the 3 months before implementation of the new delirium screening procedure and sedation scale and a 3-month period after implementation were considered for inclusion in the sample | 229 patients (128 before and 101 after) | Specific assessment tool |
| Anifantaki, 2009 | Greece | Randomised controlled trial | General ICU | To determine if a nursing-implemented protocol of daily interruption of sedative infusions vs sedation as directed by the intensive care unit team would decrease the duration of mechanical ventilation | All mechanically-ventilated patients receiving continuous infusion of sedatives 48 hours after ICU admission were eligible to be included in the trial | Ninety-seven patients were eligible to participate in the study, with 49 as intervention group and 48 as a control group | Daily interruption of sedative infusions |
| Bingham, 2010 | US | Observational Pre-post (retrospective / prospective) | General ICU | To evaluate the effectiveness of a unit-specific education intervention that emphasised hand hygiene, head-of-the-bed elevation, and oral care | The convenience sample consisted of 100 patients admitted to ICU | 100 ventilated patients not diagnosed with ventilator-associated pneumonia | Hand hygiene, Body positioning, and oral care protocol |
| Black, 2011 | Northern Ireland | Quasi-experimental design | General ICU | To study the effects of nurse-facilitated family participation in psychological care on the extent of patient delirium and psychological recovery following critical illness | Potential subjects were male or female, aged 18 years or over, with a family member who was willing to provide consent to participate | In the control group, 83 patients, in the intervention group, 87 patients | Nurse-facilitated family participation in care |
| Bliss, 2011 | US | Prospective descriptive and comparative design | General ICU | To determine the time to development, severity, and risk factors of incontinence-associated dermatitis (IAD) among critically ill patients with faecal incontinence | 18 years of age or older, admitted to one of the ICUs, and had faecal incontinence with or without urinary incontinence | 45 critically ill patients with faecal incontinence | Assessment tool |
| Blot, 2015 | Belgium | Observational cohort study | 27 ICUs in 9 European countries | To determine how the patient to nurse ratio affects the risk for ventilator-associated pneumonia | All patients who were admitted to the ICU for treatment of pneumonia or received invasive mechanical ventilation for more than 48 hours, irrespective of the admission diagnosis, were included in the initial cohort | Ventilator-associated pneumonia developed in 393 of the 1658 patients (23.7%) during their ICU stay | Nurse-to-patient ratio |
| Boev, 2012 | New York, US | Cross-sectional | Four adult ICUs | To examine patient’s perception of nursing care | All patients admitted to the critical care units during the study period was approached to complete the Patient Satisfaction Survey | A total of 1,532 patient satisfaction surveys were completed during the 5-year | Work environment |
| Boulila2016 | France | Observational Pre-post | General ICU | To assess the influence of a nurse-implemented protocol on the use of neuromuscular blockers in patients treated with 24-hour therapeutic hypothermia after out-of-hospital cardiac arrest | Patients were included in the analysis if they had been successfully resuscitated from an OHCA and admitted to our medical ICU | Among the 22 patients in the before group and the 23 patients in the after group, most were men (78%) with a median age of 66 years | Nurse-implemented protocol on the use of neuromuscular blockers |
| Casida, 2018 | US | Observational (repeated measures design) | Cardiac surgery critical-and progressive-care units | To explore the context and the influence of night-time care routine interactions (NCRIs) on night-time sleep effectiveness (NSE) and daytime sleepiness (DSS) of patients in the cardiac surgery critical-and progressive-care units of a hospital | (a) first-time elective cardiac surgery using a cardiopulmonary bypass machine; (b) male or female 18 years of age or older; (c) able to read, write, and understand written and verbal instructions in English; and (d) provide informed cons | 38 patients | Night-time care routine interactions |
| Chan, 2006 | China | Randomised controlled trial | General ICU | To determine the effect of music on physiological parameters and level of pain | Patients undergoing the application of C-clamp after PCI | Forty-three people (20 experimental and 23 control) were recruited | Music therapy |
| Chan, 2009 | China | Observational (repeated measures design) | General ICU | To determine whether definable subtypes exist within a cohort of patients listening to music concerning their physiological patterns and to compare whether associated factors vary between subjects in groups with different profiles | 1 Aged 18 year or above; 2 Understand the Cantonese dialect and/or spoken Mandarin. Literacy in Chinese preferred, but not essential; 3 Mentally alert and competent; 4 Able to communicate with the researcher by means of body gestures or in writing and 5 Without hearing defects. | 101 patients were recruited in three intensive care units in Hong Kong. | Music therapy |
| Chang, 2020 | Taiwan | Retrospective study | ICUs from two hospitals | To investigate the influence of nurse practitioners staffing on the quality of patient care in ICUs | All adult patients in the ICU who received medical treatment in 2015 | 2,932 patients | Nurse practitioner-to-patient ratio |
| Chipps, 2016 | US | Randomised controlled trial | Medical and surgical ICU | To develop an evidence-based oral care protocol for hospitalized patients and determine the impact of this protocol on health outcomes in recently extubated patients | Patients were eligible to be enrolled if they were mechanically ventilated for at least 48 hours and being considered for ventilator liberation (criteria PEEP 8 and FiO2 50%) or had been recently extubated | A total of 54 subjects completed the study: 31 in the control and 23 in the intervention | Oral Care Protocol |
| Cho, 2008 | Korea | Epidemiological | General ICU | To examine the relationship between nurse staffing and patient mortality in Korean intensive care units | The original database included all 42 tertiary and 216 secondary hospitals that operated adult ICUs | 27,372 patients from 236 hospitals (42 tertiary and 194 secondaries) | Nurse experience, Nurse staffing (staff mix, skill mix and staff ratio) |
| Cho, 2014 | Korea | Observational Pre-post (retrospective / prospective) | Medical ICU | To implement an Automatic Prediction of Delirium in Intensive Care Units (APREDEL-ICU) system to investigate its impact on nursing-sensitive outcomes and to assess nurse satisfaction with the system | (a) were > 18 years of age, (b) were not diagnosed with psychological or neurological disease, (c) had no communication problems | A total of 145 patients were involved prior to the system implementation and 172were involved after implementation | Specific assessment tool - Automatic Prediction of Delirium in Intensive Care Units (APREDEL-ICU) system |
| Cox, 1999 | UK | Observational (repeated measures design) | General ICU | To describe the physiologic and psychodynamic responses during and following the administration of therapeutic touch | Convenience sample | A total of 100 individual sessions of therapeutic touch were administered on 53 patients | Therapeutic touch |
| Cutler, 2014 | UK | Observational Pre-post (retrospective / prospective) | General ICU | To implement and evaluate the impact of oral hygiene measures (teeth brushing, 1% oral chlorhexidine and oropharyngeal suction) on the incidence of ventilator-associated pneumonia and the costs of prevention and treatment | All patients who were mechanically ventilated for at least 48 hours were included in the project except one patient in the post-change group who had experienced a previous anaphylactic reaction to chlorhexidine | Between July 2009 and December 2011, 1087 patients, mechanically ventilated for at least 48 hours and who were not chlorhexidine allergic were included in the study | Oral care protocol - Oral hygiene measures (teeth brushing, 1% oral chlorhexidine and oropharyngeal suction) |
| Dammeyer, 2013 | US | Observational (implementation) | General ICU | To provide the innovation of early mobility to ventilated intensive care unit patients | Not reported | Not reported | Nurse-Led multidisciplinary Mobility Program |
| de Araujo, 2019 | Brazil | Randomised controlled trial | General ICU | To compare the effectiveness of 2 nursing interventions in preventing dry eye in adult intensive care unit patients | Admission to the ICU, age of 18 years or older, no diagnosis of dry eye at admission, receipt of mechanical ventilation, blink rate of fewer than 5 times per minute, and a score of 7 or less on the Glasgow Coma Scale | 140 participants were randomly assigned to 1 of 2 treatment groups: a liquid artificial tears group (n = 70) and an artificial tears gel group (n = 70) | Liquid artificial tears and artificial tears gel |
| Demir, 2010 | Turkey | Randomised controlled trial | General ICU | To investigate the effect of cold application on pain and anxiety during chest tube removal | Patients hospitalized in the ICU, who had a chest tube for a duration of at least 24hours were used for this convenience sample | Ninety patients aged 18-74 years | Cold application |
| Dodson, 2014 | US | Quasi-experimental | Medical ICU | To determine whether the addition of rapid-acting insulin bolus for enteral feed coverage and a reduction in basal insulin improves glycaemic control and decrease hypoglycaemia | The population included all MICU patients for whom a physician ordered the ICU hyperglycaemia protocol | 42 adults (25 men and 17 women) were included in the pilot study, in which 17 were nondiabetic and 25 were diabetic | Nurse-led algorithm |
| Driscoll, 2017 | Australia | Systematic review | Intensive therapy units/critical care/intensive care/coronary care, high dependency, and cardiothoracic surgery units | To examine the association between nurse staffing levels and nurse-sensitive patient outcomes in acute specialist units | Patients admitted to acute specialist units, investigating the effect of nurse-to-patient ratios; Published from January 2006 to February 2017 in English; Quantitative methodology | 35 articles | Nurse-to-patient ratio |
| Dubose, 2008 | US | Prospective observational study (before/after) | General ICU | To verify if increasing input and responsibilities of ICU nursing staff in tight glycaemic control policies improves glucose control in the trauma ICU | Not reported | 291 with previous protocol; 323 after | Nurse-led algorithm |
| Elliot, 2008 | Australia | Quasi-experimental | General ICU | To improve patients’ outcomes by reducing the prevalence of pressure ulcers | The number of potential opportunities for surveying patients’ skin was 601 | A total of 563 assessments of patients’ skin was undertaken | Quality improvement projects |
| Falk, 2016 | Sweden | Retrospective study | General ICU | To investigate in a national study if the number of specialist nurses plays a role in relation to direct patient care | Not reported | 33,032 patients’ data were included | Nurse-to-patient ratio |
| Ferrie, 2007 | Australia | Observational study | General ICU | To investigate whether it is possible to reduce the incidence of diarrhoea, which should improve patient wellbeing as well as helping to reduce hospital costs | 379 consecutive tube-fed patients admitted to ICU for more than 3 days | 138 (36.41% of patients) experienced diarrhoea | Nurse-led algorithm - Bowel management protocol |
| Fraser, 2015 | US | Observational Pre-post (retrospective / prospective) | Medical, surgical, and coronary intensive care | To determine the feasibility of implementing a dedicated ICU mobility team in community hospital settings | To meet the inclusion criteria, a patient had to be a male or female adult at least 18 years of age, admitted directly to an ICU, and have an intensivist as attending or consulting physician | 66 patients were included, 66 patients received the mobility intervention | Early Mobility Program |
| Freeman, 2018 | US | Descriptive study | Cardiovascular ICU | To evaluate the effectiveness of the mobility aids | Not reported | Not reported | Early Mobility Program |
| Friesecke, 2014 | Germany | Prospective observational study (before/after) | Medical ICU | To examine whether a nurse-driven could improve early enteral nutrition of critically ill patients implementation of an existing feeding protocol | All patients were included if they were expected to stay at our 13-bed medical ICU for more than 24 h and if sufficient oral intake of food was expected to be impossible for more than 24 h | A total of 101 and 97 patients were included, respectively, before and after the intervention | Nurse-led algorithm |
| Fukuda, 2020 | Japan | Retrospective cohort study | General ICU with 10 beds | To evaluate the impact on patient outcomes of having a Certified Nurse Specialist as head nurse | Not reported | Data were collected from 3,652 people, with 1,988 in the first group (“before” group: ICU head nurse was not a Certified Nurse Specialist) and 1,664 in the second group (“after” group:  ICU head nurse was a Certified Nurse Specialist | Certified Nurse Specialist |
| Galiczewski, 2017 | US | Case-control study | Medical ICU | To determine if direct observation of the urinary catheter insertion procedure, as compared to the standard process, decreased catheter utilization and urinary tract infection rates | A convenience sampling | 140 patients that had a urinary catheter placed during their stay in the medical ICU | Direct observation |
| Han, 2010 | China | Randomised controlled trial | General ICU | To examine the effects of music intervention on the physiological stress response and the anxiety level among mechanically ventilated patients in the intensive care unit | a convenience sample of 137 patients receiving MV | 137 participants recruited 60 were men and 77 were women | Music therapy |
| Hatler, 2006 | US | Observational (implementation) | Medical ICU | To reduce rates of ventilator-associated pneumonia and catheter-related bloodstream infection | Not reported | The mean age for patients admitted to the MICU in 2005 was 55 years; 52% of the patients were male | Multidisciplinary scheduled tasks - Multidisciplinary daily rounds |
| Heslop, 2014 | Australia | Review  (Concept analysis) | General ICU | To report a concept analysis of nursing-sensitive indicators within the applied context of the acute care setting | Only primary research articles were selected | 38 journal articles | Hours of nursing care per patient day and nurse staffing (staff mix, skill mix and staff ratio) |
| Hsu, 2009 | Taiwan | Quasi-experimental | General ICU | To compare the effectiveness of three different oral care protocols in intubated patients | Age 40 years or older, oral intubation for less than 48 hours and written informed consent to participate by the patients themselves or their family members | 81 ICU patients with an oral endotracheal tube | Oral care protocols |
| Hsu, 2019 | Taiwan | Quasi-experimental | Medical ICU | To assess the effects of a back massage on improving vital signs, sleep quality, anxiety and depression among ICU patients | Convenience sampling | Sixty participants (mean age,  62⋅4±11⋅8 years) were recruited (30 in each  group) | Back massage |
| Huey-Ling, 2008 | Taiwan | Randomised, prospective cohort study | General ICU | To compare the effect of protocol-directed sedation propofol vs midazolam by nurses in intensive care | All patients received elective CABG with intubation and mechanical ventilation in CVSI | Sixty patients were assigned by randomisation table to receive propofol (n=32) or midazolam (n=28) | Protocol-directed sedation with propofol |
| Jagan, 2019 | Canada | Systematic review | General ICU | To systematically review evidence on the effects of massage on outcomes of adult critically ill patients | Primary published experimental and quasi-experimental studies reporting on the aforementioned outcome measures were included. Targeted interventions included massage alone or compared the effects of massage with other relaxation-inducing methods | 12 studies were included in the review | Massage interventions |
| Jang, 2016 | Korea | Randomised controlled trial | General ICU | To evaluate the effects of combination oral care on oral health status | Patients in an ICU who were mechanically ventilated and orally intubated | The final analysis included 18 patients with combination oral care and 17 in the control group | Oral care protocols - Tooth brushing, swabbing with 0.1% chlorhexidine and intermittent swabs of cold water |
| Karadag, 2015 | Turkey | Randomised controlled trial | General ICU | To investigate the effect of lavender essential oil on the sleep quality and anxiety level of patients in coronary ICU. | (a) 65 years of age or below, (b) diagnosed with coronary artery disease and passed the first stage of disease (the first 24–48 h of the disease), (c) no risk of heart failure and cardiogenic shock (class III and IV), (d) no history of asthma, eczema and allergies to flowers and plants, (e) not allergic to lavender, (f) communicative, no severe hearing or speech impairment, (g) no use of antidepressants, antihistamines, diuretics, hypnotics, benzodiazepines and narcotic derivatives that affect the quality of sleep and (h) willingness to participate in the research | 60 patients in coronary ICU participated in this study | Aromatherapy |
| Kelly, 2013 | US | Retrospective, cross-sectional design | General ICU | To determine whether or not the critical care nurse work environment is predictive of nurse-reported health care– associated infections | The sample included adult, non-federal, acute care hospitals that responded to the American Hospital Association Annual Survey in 2007 | 3,217 critical care nurses | Work environment |
| Kelly, 2014 | Pennsylvania, US | Cross-sectional study | General ICU | To determine the extent to which variation in ICU nursing characteristics is associated with mortality, thus potentially illuminating strategies for improving patient outcomes | Hospitals were included if they met three criteria: 1) at least 100 Medicare ICU admissions over the years 2006–2008; 2) at least five critical care nurse respondents to the nurse survey; and 3) participated in the AHA Survey. | 55,159 older adults on mechanical ventilation admitted to a study hospital | Work environment |
| Khalaila, 2011 | Israel | Observational (implementation) | General ICU | To evaluate the effectiveness and safety of a nurse-led intravenous insulin protocol designed to achieve conservative blood glucose control in patients in a medical intensive care unit | All adult patients admitted to the medical ICU during the 12 months after the initiation of the nurse-led intravenous insulin protocol, which required insulin infusion | 96 patients were enrolled and treated with the protocol | Nurse-led algorithm |
| Klein, 2018 | Ohio, US | Prospective, longitudinal, comparative study (pre-post) | Neuro ICU | To determine the sustainable impact of an early progressive mobility protocol on mobility level and clinical outcomes | All patients admitted to the neuro ICU with neurological injury | 1,117 patients during the pre-intervention (n = 260), immediate post-intervention (n = 377), and late postintervention (n = 480) periods | Nurse-led multidisciplinary mobility program |
| Knowles, 2013 | Australia | Observational study (before/after) | General ICU | To evaluate the effect of a multifaceted implementation of a bowel management protocol on outcomes for intensive care patients, in particular the incidence of constipation and diarrhoea, and on clinicians’ bowel management practices | Not reported | Pre-implementation, n = 101; post-implementation, n = 107 | Nurse-led algorithm |
| Koontalay, 2020 | Thailand | Quasi-experimental, pre-test and post-test design | General ICU | To evaluate the effects of this clinical nursing practice guideline of enteral nutrition care on the duration of a mechanical ventilator in critically ill patients to investigate whether it was able to improve clinical outcomes | (1) being conciseness; (2) aged 18 years or over; (3) vital signs stable; (4) Acute Physiology and Chronic Health Assessment II (APACHE II) ≥ 15; (5) received the enteral nutrition; and (6) willing to participate | 44 critically ill patients, the mean age of the participants was 47.11 years, ranging from 31-70 years, 39.4% were female | Clinical nursing practice guideline |
| Kram, 2015 | Maryland, US | Observational study (before/after) | General ICU | To implement the ABCDE bundle in a six-bed general adult ICU of a rural community hospital | Not reported | 47 patients in the pre-bundle group and 36 patients in the post-bundle group | Nurse-led algorithm - ABCDE Bundle |
| Krapohl, 2010 | Michigan, US | Non-experimental, correlational, descriptive design | General ICU | To determine whether the proportion of certified nurses on a unit is associated with the rate of nurse-sensitive patient outcomes | The sample population was limited to staff nurses only | 866 nurses | Proportion of nurses with specialty certification |
| Langhorn, 2015 | Denmark | Quasi-experimental | Neuro-ICU | To examine the effect of a systematic reality orientation program (RO) introduced in a neuro-intensive care unit on the duration of posttraumatic amnesia (PTA) and outcomes of patients with traumatic brain injury (TBI) | Inclusion criteria were patients of age 18Y75 years and with a Glasgow Coma Scale (GCS) score of less than 12 classified as moderate to severe during the first 24 hours and TBI diagnosed by a positive computer tomography | 33 patients in the intervention group and 43 patients in the control group were enrolled in the study | Reality orientation nursing program |
| Lawrence, 2011 | Australia | Literature review | General ICU | To critically analyse recent research that has investigated the ventilator care bundle (VCB) use, with the objective of analysing its impact on ventilator-associated pneumonia outcomes | English language experimental research published between 2004 and 2009; studies that implemented the VCB in an ICU setting; clinical outcome measures were reported | 10 research studies | Ventilator care bundle |
| Ledwith, 2010 | Philadelphia, US | Quasi-experimental, prospective repeated measures design | Neurocritical care unit | To examine the effects of patient positioning | Patients diagnosed with severe brain injury admitted to the neuro-ICU were part of this study | Thirty-three patients, including 11 women and 22 men | Body position |
| Lindgren, 2013 | Sweden | Randomised controlled trial (pilot) | Cardiovascular Surgery ICU | To evaluate the effects of touch massage on levels of anxiety and physiological stress in patients scheduled for elective aortic surgery | Patients scheduled for elective aortic surgery between October 2009 and June 2011 at Umea University Hospital were invited to participate in this study. Of 32 individuals invited, 27 agreed to participate | Twenty patients cared for in intensive care settings (mean age=65.9 years, SD=5.1) were block randomized to either a control (n=10) or an intervention (n=10) group | Massage interventions |
| Malbrain, 2010 | Belgium | Pilot prospective, single-blind randomised controlled clinical trial | Medical ICU | To compare PU outcomes in medial ICU patients nursed on either a reactive mattress overlay or an active alternating pressure mattress | Patients admitted to the ICU with a high PU risk and requiring mechanical ventilation for an estimated duration of at least 5 days either (a) with intact skin or (b) with PU’s on admission | Sixteen patients were included, eight in each group | Pressure redistributing mattress |
| Manojlovich, 2009 | Michigan, US | Cross-sectional survey | 25 ICUs | To determine the relationships between patients’ outcomes and (1) nurses’ perceptions of elements of communication between nurses and physicians and (2) characteristics of the practice environment | Not reported | 462 of 866 nurses (53.3%) completed usable surveys | Magnet-Hospital Properties Communication between nurses and physicians |
| Marklew, 2006 | Sweden | Narrative review | General ICU | To review the current published literature on the subject of positioning and gas exchange, with emphasis on oxygenation | The search was limited to ‘human’, ‘English language’ and the years 1990–2003 | Six review articles, one editorial, 12 research and clinical investigations, three reflective articles and two articles written for educational purposes | Body positioning |
| Martinez, 2017 | Chile | Prospective observational study (before/after) | Medical/ surgical ICU | To assess the efficacy and describe the implementation strategy of a multicomponent intervention to prevent delirium in an intensive care unit | Consecutive sample of critically ill patients | 287 patients, 60 in the diagnostic phase and 227 in the interventional phase | Multidisciplinary scheduled tasks - Multicomponent interventions |
| McMullan, 2013 | US | Observational | ICU different types | To describe the development and implementation of a seven-year initiative at xx hospital to decrease CLABSIs | Not reported | Not reported | Nurse-led algorithm - a central line maintenance protocol |
| Morita, 2017 | Japan | Retrospective cohort study | General ICU | To evaluate whether the presence of advanced practice nurses (APN), that is, certified nurse (CN) and certified nurse specialist (CNS) in intensive care, is associated with 30-day mortality for mechanically ventilated critically ill patients | Not reported | 45,620 patients who were admitted to an intensive care unit and received mechanical ventilation within 2 days of hospital admission between 1 April 2014 and 31 March 2015 | Nurse experience, Nurse staffing |
| Morrison, 2001 | Australia | Descriptive methodology | 93 ICUs | To identify incidents associated with nursing inexperience and estimate their effect on the quality of patient care | Seven hundred and thirty-five reports covering 1,472 incidents were identified as relating to NSI | 735 reports | Nurse experience |
| Mulkey, 2014 | Ohio, US | Pre-post quasi-experimental study | Neuroscience ICU | To assess patient characteristics and clinical outcomes of patients treated in a neuroscience Intensive Care Unit based on the level of highest mobility achieved | All adults aged 18 years and older being treated for a primary neurological injury were enrolled in the study | 228 patients | Nurse-led multidisciplinary mobility program |
| Mullin, 2011 | Connecticut, US | Descriptive study | Cardiac ICU | To describe the experience of a geriatric nurse in an ICU | Not reported | Not reported | Geriatric Resource Nurse |
| Ntoumenopoulos, 2017 | Australia | Prospective, cross-sectional study | 47 ICUs | To describe current processes of care for secretion clearance approaches within the adult, intubated and mechanically ventilated patients | All patients present in the ICU at a10 am census point on a pre-specified day in September or October 2015 | 230 patients intubated and ventilated on the study day | Secretion clearance techniques |
| Okubo, 2011 | Japan | Case-control study | General ICU | To investigate the effectiveness of the “Elevated Position” Nursing Care Program (EPNCP) in promoting the reconditioning of patients with acute cerebrovascular disease (ACD) | Acute patients hospitalized due to cerebrovascular disease who became part of the study (participants) 1 day after starting medical or surgical treatment | EPNCP group (n = 45) Control group 1 (n = 92) Control group 2 (n = 40) | Body positioning - “Elevated Position” Nursing Care Program |
| Omura, 2018 | Japan | Prospective cohort study. | Medical–surgical ICU | To investigate the frequency of post-extubation dysphagia, as diagnosed using our novel nurse-performed swallowing screening protocol, and to evaluate patient outcomes | All patients admitted to the ICU that required intubation and evaluated swallowing function after extubation | 216 patients whose swallowing functions were assessed using a novel screening protocol | Nurse-performed swallowing screening protocol |
| Orinovsky, 2018 | Israel | Case-control study | General ICU | To examine whether enteral nutrition in critically ill patients could be improved by the implementation of a nurse-led evidence-based feeding protocol | Patients’ data were included in the study if the patient was 16 years old, had received mechanical ventilation in the ICU for at least 72 hours, and was eligible to receive EN | Before (n = 65)  After (n = 52) | Nurse-Led Enteral Nutrition Feeding Protocol |
| Ozyurek, 2015 | Turkey | Randomised controlled trial | General ICU | To compare whether differences exist between 2 viscoelastic foam support surfaces in the development of new pressure ulcers | Patients older than 18 years whose expected length of stay was at least 7 days | Of 105 patients enrolled in the study, 53 were assigned to the viscoelastic foam 1 group and 52 were assigned to the viscoelastic foam 2 group | 2 viscoelastic foam support surfaces |
| Pagnucci, 2019 | Italy | Non-controlled clinical study | General ICU | To identify if complementary interventions impacted on conscious intensive care patients' perception of stress factors and quality of sleep | Conscious patients in an intensive care unit in central Italy | 74 patients | Complementary interventions for sleep-promoting |
| Papathanassoglou, 2010 | Cyprus | Narrative critical review | General ICU | To critically review evidence on the effects of psychological support during intensive care unit (ICU) treatment on adult ICU patients’ psychological and physiological outcomes | Articles containing evaluation of psychosocial interventions, either through an experimental or quasi-experimental control group design, or pre-test post-test comparisons | Fourteen studies on psychosocial support interventions were included | Relaxation and guided imagery, Massage, Music therapy |
| Papathanassoglou, 2018 | Cyprus | Randomized controlled trial (pilot) | 17-bed academic teaching general systems ICU | To investigate the effects of a multimodal integrative intervention on the incidence of pain and on secondary outcomes | more than 18 years old, understood Greek, had a score of -2 to +2 on the RASS, had a score greater than 9 on the Glasgow Coma Scale (GCS) at the time of inclusion, and had an arterial catheter in place | 60 participants (30 vs 30), the 2 groups had no statistically significant differences | Relaxation and guided imagery, Massage, Music therapy |
| Ross, 2007 | US | Pre-post quasi-experimental study | General ICU | To determine if an evidence-based practice educational programme would improve the quality of oral care delivered to mechanically ventilated patients | Patients mechanically ventilated for at least 24 h in any of the adult intensive care units were included in the sample | The pre-education sample was 52. In the post-education phase, the sample was 57 | Educational program |
| Rodríguez-Huerta, 2020 | Spain | Exploratory, two-armed, randomised, non-pharmacological, prospective study | General ICU | To evaluate whether an informative intervention by nursing professionals through Short Message Service (SMS) improved patients’ family members’ satisfaction with the intensive care experience | The participants have named contact persons of patients admitted to the ICU of any clinical specialty with a  likely stay longer than 72 hours who agreed to participate and  signed the informed consent form prepared for the study | A total of  85.3% and 75% of the intervention and control group contact persons (n=90),  respectively, were family members,  and the rest were caregivers or close relatives | Informative  intervention by nursing professionals |
| Saadatmand, 2012 | Iran | Randomized controlled trial | General ICU | To identify the effect of the nature-based sounds’ intervention on agitation, anxiety level and physiological stress responses in patients under mechanical ventilation support | The patients were aged between 18 and 65 years; were on pressure support ventilation mode and SIMV/CPAP; were able to hear; had Glasgow Coma Scale Point 9 or above | 60 patients were randomly assigned to the control (n = 30) and intervention (n = 30) groups | Nature-based sounds’ listening |
| Sales, 2011 | US | Retrospective observational cross-sectional study | 171 ICUs in 119 Veterans Health Administration hospitals | To assess the association of some of these factors with in-hospital mortality among patients who have an ICU admission during their hospital stay | Not reported | 34,093 patients | Hours of nursing care per patient day and nurse staffing (staff mix, skill mix and staff ratio) |
| So, 2019 | China | Quasi-experimental study design | A 22-bed mixed medical and surgical ICU | To evaluate the effects of a nurse-led critical care follow-up program on ICU readmission and hospital mortality in patients with respiratory problems | (1) Adult patients 18 years  old; (2) first ICU admission and staying in the ICU for at least 24 h; and (3) having at least one respiratory problem | A total of 369 participants (the intervention group: 185; the control group: 184) | Nurse-led, multidisciplinary ICU follow-up program |
| Sosnowski, 2015 | Australia | Systematic literature review | General ICU | To appraise current research which examines the impact of early rehabilitation practices on functional outcomes and quality of life in adult intensive care unit (ICU) survivors | Only studies that related to the ABCDE bundle and PAD guideline were sought | 10 studies included in the synthesis | Early mobility program |
| Stalpers, 2015 | The Netherlands | Systematic review | General ICU | To systematically review the literature on relationships between characteristics of the nurse work environment and five nurse-sensitive patient outcomes in hospitals | Included were quantitative studies published from 2004 to 2012 | 29 studies were included in the review | Work environment |
| Stone, 2007 | Pennsylvania, US | Correlational cross-sectional | General ICU | To examine the effects of a comprehensive set of working conditions on elderly patient safety outcomes in intensive care units | Not reported | 15,846 patients in 51 adult intensive care units in 31 hospitals depending on the outcome analysed; 1095 nurses were surveyed | Staffing and organisational climate |
| Stuck, 2011 | US | Narrative review | General ICU | To promote consideration of a patient-centred approach to modifying one of the suspected risk factors for ICU delirium and sleep deprivation | Not reported | Not reported | Patient-centred approach to reducing sleep disruption |
| Su, 2012 | Taiwan | Randomised controlled trial | Medical ICU | To examine the effects of non-commercial music on quality of sleep and relaxation indices, including heart rate, mean arterial pressure, and respiratory rate in patients in intensive care units | (1) age>18 years old; (2) APACHE II score 25; (3) ability to communicate in either Mandarin or Taiwanese; (4) being conscious and clear; (5) having a length of residency in the ICU for more than 24 hours; (6) having an arterial catheter inserted | 28 patients aged 39–78 years were randomly assigned to music and control groups | Music therapy - listening to non-commercial music |
| Sutton, 2016 | New Zealand | Descriptive post-implementation | 18-bed general ICU | To describe a nurse-initiated quality improvement (QI) project that improved the care of critically ill patients | Not reported | Comparison of audit data collected in 2014 and 2015 | Nurse-initiated quality improvement (QI) project |
| Swadener-Culpepper, 2008 | Georgia, US | Pre-post quasi-experimental study | General ICU | To determine the impact of continuous lateral rotation therapy (CLRT) on patients considered to be at high risk for pulmonary complications. | Patients at risk for pulmonary complications as defined by PaO2/FiO2 ratio < 300, FiO2 > 50% for more than 1-hour, positive end-expiratory pressure ≥ 8, or a Predicus score of ≥ 5 | Ninety-five patients met the study criteria from January 1, 2003, to May 1, 2004 | Lateral Rotation Therapy |
| Tracy, 2011 | Minnesota, US | Narrative review | General ICU | To describe nonpharmacological interventions to manage common symptoms in patients receiving mechanical ventilation | Not reported | Not reported | Nonpharmacological complementary therapies |
| Wanik, 2019 | Connecticut, US | Observational (implementation) | Medical and surgical ICUs | To evaluate the efficacy of implementing protocols to decrease constipation, diarrhoea, and inappropriate testing for hospital-onset C difficile infection, and to deliver larger enteral nutrition volumes | A prospective convenience sample | A total of 43 patients: 23 in the pre-protocol group and 20 in the post-protocol group | Bowel protocols |
| Winkelman, 2012 | Ohio, US | Quasi-experimental | Medical or surgical ICU | To quantify the effects of the nurse-initiated mobility exercise protocol according to outcomes | Subjects were assessed for enrolment if they experienced more than 48 hours of mechanical ventilation and were anticipated to continue receiving mechanical ventilation for the next 24 hours | 75 patients form the sample | Nurse-led multidisciplinary mobility program |
| Yeh, 2003 | Taiwan | Cross-sectional | 11 adult ICUs | To explore the roles of nursing care on the occurrence and consequences of unplanned endotracheal extubation | Not reported | Over 18 months, 1176 patients were intubated, and 265 incidents of unplanned endotracheal extubation occurred | Nurse experience, Nurse-to-patient ratio |
| Zhang, 2017 | China | Prospective before-after study | Cardiothoracic ICU | To determine whether a nursing intervention targeting risk factors could decrease the incidence of POD among patients who had coronary artery bypass grafting | Eligible patients were 18 years old or older. They were included in the study, when they stayed in the ICU for more than 24 | A total of 278 patients were included in the study 137 in Control Group and 141 in Intervention Group | Nursing protocol |
| Zurmehly, 2012 | Ohio, US | Quasi-experimental design | 12-bed hospital ICU | To evaluate the effectiveness of a nursing quality education improvement program on oral care practice in reducing the incidence of ventilator-associated pneumonia within the ICU | Patients who had used a mechanical ventilator for more than 48 hours but did not have ventilator-associated pneumonia at the time of admittance to the ICU were included in the study | 44 nurses completed the educational program | Oral care protocols |

Abbreviations: UK, United Kingdom; US, United States; ICU, Intensive Care Unit.
